# Majority Decision-Making Works Best Under Conditions of Leadership Ambiguity and Shared Task Representations

**DOI:** 10.3389/fpsyg.2021.519295

**Published:** 2021-06-14

**Authors:** Michaéla C. Schippers, Diana C. Rus

**Affiliations:** ^1^Department of Technology and Operations Management, Rotterdam School of Management, Erasmus University, Rotterdam, Netherlands; ^2^Department of Organizational Psychology, Faculty of Behavioural and Social Sciences, University of Groningen, Groningen, Netherlands

**Keywords:** group decision-making, decision rules, shared task representations, leadership ambiguity, team performance

## Abstract

The effectiveness of decision-making teams depends largely on their ability to integrate and make sense of information. Consequently, teams which more often use majority decision-making may make better quality decisions, but particularly so when they also have task representations which emphasize the elaboration of information relevant to the decision, in the absence of clear leadership. In the present study we propose that (a) majority decision-making will be more effective when task representations are shared, and that (b) this positive effect will be more pronounced when leadership ambiguity (i.e., team members’ perceptions of the absence of a clear leader) is high. These hypotheses were put to the test using a sample comprising 81 teams competing in a complex business simulation for seven weeks. As predicted, majority decision-making was more effective when task representations were shared, and this positive effect was more pronounced when there was leadership ambiguity. The findings extend and nuance earlier research on decision rules, the role of shared task representations, and leadership clarity.

## Introduction

*“When exploring the Northwest Territory in 1805, Captain Clark used the majority rule to decide where to set his winter camp (Ambrose, 1996; Moulton, 2003). Everyone in the expedition, including servants and native guides, had an equal vote in the majority rule decision.”*

– (Hastie and Kameda, 2005, p. 506).

Organizations nowadays often rely on teams when making decisions that require a wide array of knowledge (Dooley and Fryxell, 1999; Kozlowski and Bell, 2003). The effectiveness of those decision-making teams is for a large part dependent on their ability to make use of and integrate information successfully (e.g., van Ginkel and van Knippenberg, 2008; Schippers et al., 2013) and on the decision rules they apply (Stasser et al., 1980; Nitzan and Paroush, 1985; Hastie and Kameda, 2005). One of the most prevalent decision rules that teams use to make decisions is majority decision-making (Baron et al., 1992), which typically relies on pooling preferences and making compromises among team members. The prevalence of the majority rule can be explained by its transparency, ease of execution, and its appeal to people’s innate sense of justice (Hastie and Kameda, 2005). Yet, to date, our understanding of the relationship between majority decision-making and team performance is limited. For instance, some studies have shown that it can be efficient and ensure quick decision-making (cf. Hare, 1976; Kerr et al., 1976), yet others have found mixed effects on decision-making effectiveness, especially in complex interdependent tasks that require the discussion and integration of uniquely held information within the team (cf. Beersma and De Dreu, 2002; Ten Velden et al., 2007; van Ginkel and van Knippenberg, 2008). Given the prevalence of this decision-making rule in teams working on complex interdependent tasks, it is important to understand under what conditions it may lead to better quality decisions and better team performance.

The majority decision-making rule is predicated on the notion that a team will reach better decisions if the preferences of individual team-members are democratically pooled and integrated into the final decision. Yet, for this democratic pooling to lead to a good quality decision, it is important that individual members’ preferences are based on an as full and accurate understanding of task-relevant information as possible. In complex interdependent tasks, this would require that uniquely distributed information surfaces during group discussion, is elaborated upon and successfully integrated into the final decision via voting. Yet, numerous studies have shown that groups often fail to exchange information (cf. Gruenfeld et al., 1996; Wittenbaum and Stasser, 1996; Wittenbaum et al., 2004), and even if they do, they often do not elaborate on and integrate this information into their final decisions (Gigone and Hastie, 1993; for a meta-analysis see Mesmer-Magnus and DeChurch, 2009; for reviews see van Ginkel and van Knippenberg, 2012; Schippers et al., 2014). Moreover, this failure to discuss and integrate information, may be compounded in teams using a majority rule. Focused on quickly pooling preferences, team members may fail to discuss the underlying assumptions behind their preferences (Mohammed and Ringseis, 2001). In addition, uniquely held information may be less likely to surface since dissenting minorities may fail to voice their concerns (Stasser and Birchmeier, 2003). In this respect, prior research has shown that shared task representations – i.e., the shared realization that the task needs information elaboration – play an important role in facilitating the effective use of informational resources in groups. To this end, they have been shown to facilitate information sharing and critical discussion (Postmes et al., 2001), the voicing of different opinions (even, minority ones), and a more thorough elaboration and integration of the information at hand (cf. Ten Velden et al., 2007). Hence, the extent to which teams have developed shared task representations may play an important role in facilitating or hindering the integration of relevant information into the final decisions of teams favoring a majority rule. Therefore, we argue that shared task representations moderate the relationship between majority decision-making and team performance. Specifically, we expect that for teams favoring a majority rule, high (vs. low) levels of shared task representations will be positively (vs. negatively) associated with team performance.

Another factor that may facilitate or hinder the extent to which team members exchange and process information is the extent to which there is clarity about who is responsible for leadership in the team. In prior research, clarity of leadership – that is, team members’ shared perceptions *“about the extent to which leadership roles are clear within the team”* (West et al., 2003, p.395) – has been associated with improved team effectiveness (for a review see Smith et al., 2018). Indeed, a clear understanding about who is responsible for taking the lead can serve a critical role in helping teams exchange, process and coordinate the integration of information. However, for teams who choose to use an egalitarian majority rule, the presence of one clear leader may shift the burden of responsibility from the team to the leader and may make the team more vulnerable to a premature “closing of the group mind” (Kruglanski and Webster, 1991; De Grada et al., 1999; Tetlock, 2000). In addition, one clear leader may have a disproportionate impact on the decision by swaying team members’ preferences in a certain direction (e.g., Janis, 1972, 1982). Hence, in the presence of a clear leader, the effects of shared task representations on team performance may be less pronounced for teams using a majority rule, since a leader may either facilitate or hinder the extent to which team members exchange, process and integrate information. On the other hand, a lack of clear leadership – or leadership ambiguity as we will call it henceforth (cf. West et al., 2003; Carson et al., 2007) – may create a context that amplifies the potential effects of shared task representations on team performance for teams using a majority rule. Specifically, teams with high shared task representations using a majority rule may benefit from leadership ambiguity. These teams have developed a shared understanding that success is predicated on the exchange, discussion and integration of diverse information. In the absence of a clear leader, team members might be more likely to have a sense of shared responsibility for team outcomes and might be more motivated to share and thoroughly discuss relevant information. In turn, team members’ preferences would be shaped by relevant information rather than by a leader’s opinion, which should translate into better performance with a majority decision-making rule. On the other hand, leadership ambiguity may be particularly harmful for teams with low shared task representations using a majority rule. Without a shared understanding that success depends on information exchange and with no clear leader to potentially facilitate information sharing, team-members ‘preferences are likely to be based on incomplete and/or biased information. This, in turn, should result in less-than-optimal decisions with a majority rule. In short, we argue that leadership ambiguity will amplify the positive (vs. negative) effects of shared task representations on team performance under conditions of high majority decision-making.

The current study makes a number of contributions to the literature on team decision-making and the broader team performance literature. First, it puts two understudied concepts – majority decision-making and leadership ambiguity – center-stage in the study of team decision-making, and does so in the controlled context of a management simulation. Second, it points to the importance of shared task representations for team performance on complex interdependent tasks, which require the sharing, discussion and integration of information. Finally, by focusing on the interactive effect of majority decision-making rules, shared task representations, and leadership ambiguity on team performance, it shows that it is the combination of these three factors that determines group outcomes, rather than the isolated effects of any of these variables.

## Theoretical Background and Hypotheses

### Majority Decision-Making and Team Performance

Previous research suggests that groups are likely to reach higher quality decisions if they are able to share, discuss and integrate information and that this is all the more important for teams whose members are interdependent and need to make decisions based on unique information distributed within the team (cf. Scott and Kameda, 2000; van Ginkel et al., 2009; van Ginkel and van Knippenberg, 2012; Schippers et al., 2014). Indeed, for distributed information to be used effectively, it would require that it surfaces during group discussion, is carefully elaborated on and successfully integrated into the final decision (Schippers et al., 2007; De Dreu et al., 2008; Homan et al., 2008). However, the likelihood that this will happen will be contingent on the decision-making procedures or rules that teams apply. These decision rules affect the way teams make decisions and, therefore, may help or hinder information exchange and processing (cf. Hastie and Kameda, 2005; Bianco et al., 2006).

A group decision rule specifies how decisions are made within a team, and can be defined as *“a rule that specifies, for any given set of individual preferences regarding some set of alternatives, what the group preference or decision is regarding the alternatives”* (Miller, 1989, p. 327). The two rules most often used in groups are the majority rule and the unanimity rule (Hare, 1976; Miller, 1989; Baron et al., 1992), although it is also conceivable that a directive team leader or dominant group member makes most of the decisions (cf. Van de Ven and Delbeco, 1971; Leana, 1985). Importantly, these rules set the context for the extent to which information is likely to be discussed and integrated in the final decision. For instance, unanimity requires agreement from all team members, therefore, group decisions may require more discussion, may be harder to reach and may integrate more diverse points of view in the final decision (e.g., Castore and Murnighan, 1978; Miller, 1989). In contrast, if a dominant group member takes the lead and makes most of the decisions, it is likely that group decisions may require little discussion, may be easier to reach and may fail to integrate diverse perspectives in the final decision (cf. Leana, 1985). Finally, the most prevalent rule (Kameda et al., 2002; Kameda and Tindale, 2006) used in groups — the majority rule— relies on the democratic pooling of preferences from different group members. This decision-making rule, based on shared preferences has been shown to provide a “fast and frugal” heuristic in complex decision environments (Hastie and Kameda, 2005) and to lead to more efficient and less time-intense decision-making (Hare, 1976; Kerr et al., 1976).

However, it is also vulnerable to information-processing failures, since decisions may be based on biased or incomplete information, especially in tasks that require the exchange and integration of information from multiple perspectives. For instance, prior research has found that a majority decision-making rule is susceptible to agenda setting and other forms of strategic behavior (cf. Bianco et al., 2006), especially in situations where there are misaligned interests, which could be resolved by negotiation (e.g., Mohammed and Ringseis, 2001; Ten Velden et al., 2007). To this end, experimental research among 97 three-person groups in a negotiation situation showed that under a majority rule, pro-self-oriented majority members coalesce at the expense of the minority (Ten Velden et al., 2007). Thus, in situations where interests are misaligned a majority decision-making rule may lead to strategically biased decisions. Research has also found that, in situations where interests are aligned and team members strive for the same collective outcome, a majority rule may induce team members to behave in the group interest (e.g., Kameda et al., 2002; Kameda and Tindale, 2006). Yet, even in situations where teams have aligned interests, team decision-making may still be based on incomplete information. Being motivated to behave in the interest of the group does not guarantee that uniquely distributed information will surface in the discussion and that it will be integrated in the final decision (cf. Winquist and Larson, 1998; Kerr and Tindale, 2004; Nijstad and De Dreu, 2012). For instance, interdependent teams focused on pooling preferences may fail to discuss the underlying assumptions behind their preferences (Mohammed and Ringseis, 2001), which may lead to lower quality decisions based on incomplete or distorted information. Moreover, critical yet uniquely held information may be less likely to surface, especially if there are conformity and time pressures within the team (cf. Schippers et al., 2014) as team members holding a minority preference may not voice their opinions (Stasser and Birchmeier, 2003). Importantly, these failures to share and integrate distributed information into decisions, may have a compound effect on team performance, if teams have to make multiple interrelated decisions in complex business environments within a short time period, such as, for instance, in business simulations (e.g., Hung and Ryu, 2008; Mathieu and Rapp, 2009; De Leeuw et al., 2015). Therefore, we argue that, in complex interdependent tasks, a majority decision-making rule might be negatively related to team performance.

*Hypothesis 1:* Majority decision-making will be negatively related to team performance.

### Majority Decision-Making and Team Performance: The Moderating Role of Shared Task Representations

However, the relationship between majority decision-making and team performance might not be as straightforward, since the effectiveness of the decision rule seems to be largely contingent on the extent to which team members discuss and integrate uniquely held information within the team (cf. Mohammed and Ringseis, 2001; Beersma and De Dreu, 2002; Kerr and Tindale, 2004; Ten Velden et al., 2007). Given that some teams are better than others at discussing and integrating this information, it is likely that factors which facilitate information elaboration and integration may serve as important moderators of the relationship between a majority decision-making rule and team performance. To this end, prior research has shown that shared task representations play an important role in facilitating the effective use of informational resources in groups (van Ginkel and van Knippenberg, 2008). Shared task representations entail a common understanding among the team members that the task needs information sharing, elaboration and integration (van Ginkel and van Knippenberg, 2008; van Ginkel et al., 2009). As such they can be conceptualized as a kind of team mental model concerning how to deal with information (Cannon-Bowers et al., 1993; Marks et al., 2000; Kerr and Tindale, 2004; Mathieu et al., 2005). It is worth noting though, that, shared task representations (i.e., the realization that information should be elaborated on) are an antecedent to information elaboration, and, while highly correlated, they are not necessarily the same (van Ginkel and van Knippenberg, 2008). Prior research has, however, shown that shared task representations facilitate the voicing of different opinions (even, minority ones) and a more thorough elaboration and integration of the information at hand (cf. Ten Velden et al., 2007). In addition, a study by Kilduff et al. (2000) found that teams that had managed to develop a shared understanding of what contributes to organizational success and failure performed better than their counterparts who failed to do so in a management simulation. Since the performance of teams using a majority rule will largely hinge on whether uniquely distributed information will surface and be integrated into the final decisions, we argue that the extent to which they have developed shared task representations may either facilitate or hinder team performance.

Teams differ in the extent to which they recognize the need for information elaboration and develop shared task representations (cf. Nijstad and De Dreu, 2012; Schippers et al., 2013). Yet, for teams using a majority decision-making rule, it is especially important to have critical thought norms (Postmes et al., 2001), such as shared task representations, that facilitate the integration of information and ensure informed decision-making (cf. Kerr and Tindale, 2004; Nijstad and De Dreu, 2012). Therefore, we argue that the extent to which teams have developed shared task representations may be especially important in facilitating the discussion and integration of relevant information into the final decisions of teams favoring a majority rule. Specifically, we expect that teams with high levels of shared task representations will benefit from a majority decision-making rule, since team members will be more inclined to voice and defend their ideas and findings (even if they are different from the majority) and will take more trouble to elaborate on and integrate the information at hand. In contrast, we expect that the performance of teams with low levels of shared task representations is likely to suffer from a majority-decision making rule, since team members might be more inclined to focus on efficiently pooling preferences and reach quick decisions, thereby failing to integrate vital information into the final decision. In sum, we expect that a majority decision-making rule and shared task representations will interact in predicting team performance. Specifically, we predict that:

*Hypothesis 2:* Shared task representations moderate the relationship between the extent of majority decision-making and team performance, such that when:
  (*a*) shared task representations are *high* the relationship between majority decision-making and team performance is *positive.*
  (*b*) shared task representations are *low* the relationship between majority decision-making and team performance is *negative.*

### Majority Decision-Making and Team Performance: The Moderating Role of Shared Task Representations and Leadership Ambiguity

Another factor that may facilitate or hinder the extent to which team members voice their opinions and integrate critical yet uniquely held information into their decisions is the extent to which there is clarity about who is responsible for leadership in the team. Leadership has often been proposed to be crucial for team effectiveness (Hackman, 1990; Cohen and Bailey, 1997; Carson et al., 2007), and some have argued that it is *the* most critical ingredient (Sinclair, 1992; Zaccaro et al., 2001). In this respect, most leadership research has focused on the effects of a single formally appointed leader on team processes and performance, with some more attention having been paid in recent years to other forms of leadership, such as emergent leadership (e.g., Taggar et al., 1999; Cogliser et al., 2012; Yammarino, 2012) and shared/distributed leadership (Carson et al., 2007; for reviews see Pearce and Conger, 2003; Pearce and Manz, 2005; D’Innocenzo et al., 2014; Sun et al., 2016). Whereas these lines of inquiry have been important in furthering our understanding of how different types of leadership may affect team performance, they ignore the fact that teams may naturally differ in the extent to which there is clarity about who is responsible for leadership in the team.

Previous research has introduced the concept of leadership clarity to refer to the “*shared perceptions of group members about the extent to which leadership roles are clear within the team*” (West et al., 2003, p. 395). In this research, we will rely on the West et al. (2003) definition to conceptualize leadership ambiguity as the shared perceptions of team members that there is no clear team leader. Leadership ambiguity in a team might exist for a number of different reasons. For instance, in the absence of a formally assigned leader, there might be multiple individuals who informally take charge at different points in time and/or on different tasks, yet they are not seen by other team members as having a team leadership role. Thus, what is important, is that team members share a common perception that there is no single overall team leader. Overall, higher levels of clarity regarding team leadership have been associated with improved team innovation and effectiveness (for a review see West et al., 2003; Smith et al., 2018), and it appears that, in general, teams are less likely to be successful when they have no clear leader (Cohen and Bailey, 1997). Indeed, a clear understanding about who is responsible for taking the lead can serve a critical role in helping teams exchange, process and coordinate the integration of information. Yet, research has also shown that team performance on complex tasks can suffer if a clear leader dominates the discussion, states their opinion early on in the decision-making process, and eliminates dissenting opinions (Janis, 1972, 1982; Anderson and Balzer, 1991; Taggar and Seijts, 2003). In addition, evidence from a few related lines of research seems to suggest that, indeed, teams working on complex interdependent decision-making tasks where there is a high need to integrate distributed information, might benefit from not having *one* clear leader. For instance, research on shared leadership *(“an emergent and dynamic phenomenon whereby leadership roles and influence are distributed among team members”*; D’Innocenzo et al., 2014, p. 5) suggests that this form of leadership is positively related to team innovation (Hoch, 2013) and performance, especially in teams that have a shared purpose and norms favoring social support and voice (Carson et al., 2007). In addition, research on self-managed teams suggests that, due to the increased flexibility and adaptability afforded by the absence of a formal leader, they can be effective, especially if they manage to sidestep dysfunctionalities arising from conflict (Langfred, 2000, 2007). Granted, shared leadership and the absence of formal leadership in self-managed teams are not conceptually the same as leadership ambiguity. In the context of shared leadership, different people have a clear leadership role, while leadership ambiguity is about the shared perception that there is no clear group leader. Yet, findings from these research streams do suggest that, under certain conditions, the absence of a single clear leader (since leadership roles emerge and shift dynamically over time) may facilitate the discussion and more thorough elaboration of distributed information, and, in turn, translate into better performance for teams working on complex interdependent tasks.

Thus, we propose that, for teams using a majority rule –whose success is predicated on team members sharing and discussing uniquely held information and integrating it into a final decision – leadership ambiguity may create a context that amplifies the potential effects of shared task representations on team performance. Specifically, we expect that high leadership ambiguity should strengthen the positive (vs. negative) effects of shared task representations on team performance under conditions of high majority decision-making and we will explain our reasoning below. We have previously argued that teams with high shared task representations, using a majority rule, should be more inclined to voice their ideas and take more trouble to elaborate on and integrate distributed information. Yet being inclined to share and discuss information does not guarantee that information will be shared or integrated into the final decision. Even in these teams, a clear team leader might dominate the discussion, eliminate dissenting opinions (cf. Janis, 1972, 1982; Anderson and Balzer, 1991; Taggar and Seijts, 2003), and cause a premature “closing of the group mind” (Kruglanski and Webster, 1991; De Grada et al., 1999; Tetlock, 2000; Pierro et al., 2003), thereby preventing thorough information elaboration. Furthermore, even if information is thoroughly elaborated on, a clear leader may have a disproportionate impact on the decision by swaying team members’ preferences during the voting process (e.g., Janis, 1972, 1982), thereby offsetting the potential positive impact of shared task representations on team performance. Hence, in the presence of a clear leader, the positive effects of shared task representations on team performance may be less pronounced for teams using a majority rule, since a leader may hinder the extent to which team members exchange, process and integrate information. In contrast, high leadership ambiguity should create an especially favorable environment for the decision-making quality of teams with high shared task representations using a majority rule. The shared perception among team members that there is no clear team leader combined with information elaboration norms, such as high task representations, may free individual team members from any potential conformity pressures induced by a dominant group member, increase felt responsibility for the final outcome, and motivate and enable them (Wittenbaum et al., 2004) to share and discuss distributed information. This, in turn, would ensure that high shared task representations actually lead to a more thorough elaboration of information, thereby leading to voting preferences shaped by relevant information (rather than by a dominant team member’s opinions), which should translate into better performance. On the other hand, leadership ambiguity may be particularly harmful for teams with low shared task representations using a majority rule. Without a shared understanding that success depends on information exchange and with no clear leader to potentially facilitate information sharing, team-members ‘preferences are likely to be based on incomplete and/or biased information. This, in turn, would prevent teams from discussing and integrating vital information into their final decisions and should translate into poor performance.

**FIGURE 1:**
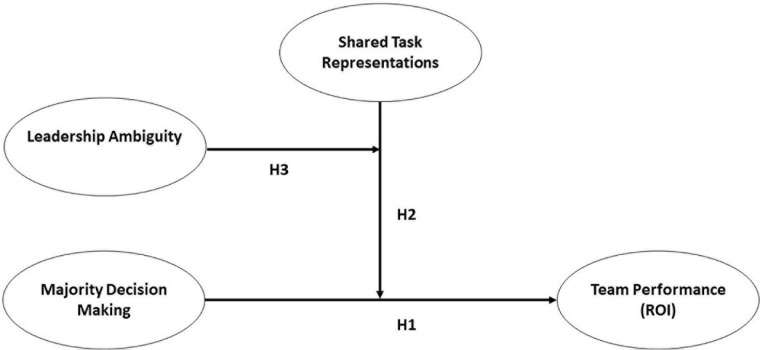
Research model of hypothesized links between majority decision-making, shared task representations and team performance in the game (ROI). Note that the hypotheses build upon each other, H3 represents a three-way interaction model of team performance (ROI).

In sum, we argue that majority decision-making, shared task representations, and leadership ambiguity interact in predicting team performance on complex interdependent tasks (see Figure 1 for our complete research model). Specifically, we expect high leadership ambiguity to strengthen the positive (vs. negative) effects of shared task representations on team performance under conditions of high majority decision-making. Under conditions of low leadership ambiguity, we expect the effects of shared task representations on team performance to be less pronounced for teams using a majority rule, since the presence of a clear leader may either compensate for low shared task representations by taking on a coordinating function or may hinder the effective use of high shared task representations by dominating team processes. Do note that in our hypothesis, we make specific *a priori* predictions regarding the expected pattern of the slopes under conditions of high leadership ambiguity and not under conditions of low leadership ambiguity.

*Hypothesis 3:* Shared task representations and leadership ambiguity will jointly moderate the relationship between the extent of majority decision-making and team performance, such that:
  (*a*) when shared task representations are *high*, combined with *high* leadership ambiguity, the relationship between majority decision-making and team performance will be *positive;*
  (*b*) when shared task representations are *low*, combined with *high* leadership ambiguity, the relationship between majority decision-making and team performance will be *negative.*

## Methods

### Sample and Procedure

Data for this study were collected by means of an online survey handed out to all team members as part of a larger investigation involving teams taking part in a supply chain business simulation. As such, the study involves both subjective and objective measures, whereby our predictors consist of subjective perceptions of team processes, whereas our outcome variable consists of an objective measure of team performance. The initial sample consisted of a total of 376 individuals, distributed over 94 four-person teams, who participated in the simulation either on a voluntary basis or as part of a supply chain management course. Participants had direct or indirect experience in supply chain management and the majority were employed professionals, such as general managers, operational managers, financial managers, and supply chain managers. A small minority of participants consisted of supply chain management students. The response rate for the online survey measuring perceptions of team processes was 83% (258 persons from 82 teams). One team was removed from the analysis, due to their low participation during the game, as a result of which the team did not receive scores on the dependent variables. For teams to be included in the final dataset, at least two of the four team members had to have completed the survey. This resulted in a final sample that consisted of 254 persons distributed over 81 teams. Of these respondents, 76.4% were male and the average age was 33.7 years (SD = 9.42). 81.5% of the respondents were Dutch nationals, the remaining respondents were American (18.5%); 39.8% of the respondents had at least a bachelor’s degree and 2.7% had another advanced degree or professional qualification.

### The Simulation

In the operations management domain, games and simulations represent an important learning tool for learning the intricacies of team and cross-functional decision-making (Sweeney et al., 2010). The ‘‘Fresh Connection’’ business simulation, which was used in this study, requires members to work as an integrated sales and operations team^1^. Teams played the game by competing with other teams, yet their own performance was not dependent on those other teams. The game has some similarities to the “Beer Game” (Goodwin and Franklin, 1994; see also Gino and Pisano, 2008), although in this particular game the participants were expected to run the whole company, with an emphasis on the supply chain (De Leeuw et al., 2015). As such, it is richer and more complete than most other games, such as the beer game which is only aimed at the distribution side of the supply chain. The interactive, computer-based simulation was an ongoing experiential exercise for professionals working in the field, and was based on events in the production and supply of fresh juices to customers. In the simulation, participants learned that the Fresh connection products, such as fruit juices, are stored in pallets in the finished goods warehouse. The products have a shelf life of 20 weeks, and stay in the warehouse, until a delivery is made, or the shelf life expires. Local and regional suppliers deliver the raw materials, and concentrated fruit juice is acquired from fruit traders. A decision-making team (consisting of four members) has to consider various issues such as its sales and operations plan for the purchasing of supplies, demand forecasting, product management, pricing, promotions, delivery lead times, capacity planning (including decisions involving the number of shifts, overtime, scheduled maintenance), production planning, and inventory planning. Within each team, there were four different roles: a supply chain vice-president (responsible for supply chain strategy and control decisions), a purchasing vice-president (responsible for the choice of suppliers, supplier agreements etc.), an operations vice-president (concentrating on the organization of operations and the warehouse), and a sales vice-president (responsible for decisions on customer service, the priorities of orders, and promotional activities). As such, the sales and operations planning process in this simulation is key to company success and encompasses more than only the supply chain department (De Leeuw et al., 2015). During the game, team members received unique information relevant to their role and it was important to share this unique information with all team members. Although most teams passed on the information received in the emails to other team members, the extent to which the information led to the development of shared task representations and was actually processed and elaborated upon varied across teams.

**FIGURE 2:**
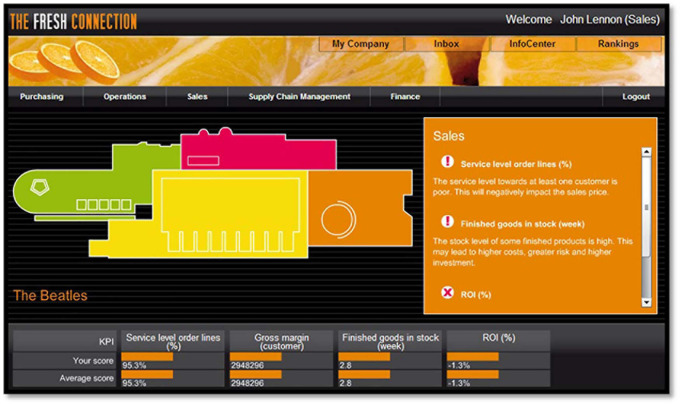
Screenshot of “Fresh Connection” business simulation.

Participants were expected to run the company for seven decision periods of one week each, that is, seven rounds, where each week actually represented six trading months for the company in the game. The game started with a video message from the former CEO, who explained current issues in the company. Teams participating in the research received feedback on their team level scores and on the meaning of these measures, and all teams received detailed feedback reports (for an elaborate description of the game see De Leeuw et al., 2015). The simulation was highly realistic, was related to actual work settings, and had high dynamic and coordinative complexity (see also Seijts et al., 2004). Care was taken to ensure the realism of the simulation, including role descriptions, background information, graphics, pictures, e-mail simulation, organizational charts, and interactive activities. During the game, individual team members received e-mail messages with unique information related to their role and the whole team also received e-mails about various events and developments such as new clients, delivery problems, special customized products, etc. Thus, teams were expected to integrate and make sense of all this information (i.e., unique information held by team members and shared information held by the whole team) in order to make choices and reach decisions (for a screenshot of the game, See Figure 2). Numerous decisions had to be made while playing the game, and trade-offs were implied in every decision. Team decisions were uploaded and processed and the simulation then provided a weighted team-performance composite for each round. The extent to which teams were able to balance these trade-offs, determined their performance (ROI).

### Measures

After the participants had completed the game, but before they received feedback on their final performance, they completed a survey that measured various team processes (see Appendix for all items used in the survey).

#### Shared Task Representations

Five items based on van Ginkel and van Knippenberg (2008) were used to measure the degree to which team members had developed a shared realization that the task needs sharing of distributed information, critical discussion and information elaboration, and the integration of this information into the final decision. The items were slightly adapted to fit the context of the game. An example item is “For high quality performance it was important to base the decision on as much information as possible” (1 = *strongly disagree*, 5 = *strongly agree*, α = 0.61, *F* = 1.61, *p* < 0.01; ICC(1) = 0.16, ICC(2) = 0.61, rwg_(j)_ = 0.92).

#### Majority Decision-Making

Based on prior literature (e.g., Bianco et al., 2006; Ten Velden et al., 2007), we developed a one-item measure with three different answer options to identify the decision-making rule that was used in the teams. Respondents were asked “*How were decisions made in your team?*” and they could answer by selecting *only one out of three* answer options indicating whether their team used a majority rule, a unanimity rule, or had one dominant member making decisions (see Appendix for the answer options). For each respondent, the chosen answer option was coded as 1 and the two options that were not chosen were coded as 0. Majority decision-making was calculated to represent the proportion of team members indicating that a majority decision-making rule was used in their team. For example, if 2 team members in a four-member team selected ‘‘We had a majority rule’’ (coded as 1), majority decision-making was 50%^2^.

#### Leadership Ambiguity

We used the same one-item measure with several answer options developed by West et al. (2003) to assess leadership ambiguity (in their research it was called lack of leadership clarity). Each respondent was asked “*To what extent is there an overall leader/coordinator in your team?”* and they could answer by selecting *only one out of five* answer options indicating the extent to which there was an overall team leader (see Appendix for the answer options). For each respondent, the chosen answer option was coded as 1 and the four options that were not chosen were coded as 0. We followed the same procedure as West et al. (2003) to calculate leadership ambiguity. Specifically, leadership ambiguity was calculated to represent the proportion of team members that indicated that ‘‘There is no clear leader/coordinator’’^3^. For example, if three team members in a four-member team selected “There is no clear leader/coordinator” (coded as 1), leadership ambiguity was 75%.

#### Team Performance

Team performance in the simulated game was assessed by the team score of Return on Investment (ROI) of the fictitious company. The objective for each team was to achieve the best return on investment (ROI). It was not only crucial to make as much money as possible, but also to manage investments in a proper way (see also De Leeuw et al., 2015). As each round represented a decision horizon of six months, the focus of the game was on strategic and tactical supply chain decisions (for a screenshot of the game, see Figure 2). After each round, participants could see their own performance and compare it with other teams’ performance in the competition. During each round, players made progressively more difficult decisions, as complexity was gradually added in each round. Thus, it was key for teams to choose a strategy and to make decisions in accordance to the chosen strategy. Furthermore, performance in each round was calculated independently, and teams did neither suffer negative consequences nor reap benefits resulting from poor or very good decisions made in earlier rounds (De Leeuw et al., 2015).

The simulation automatically calculated a team’s overall score by indexing each factor on a scale of -1 to 1, according to the team’s relative performance in the simulation. The final score represented a weighted average of the score over six rounds, where the last two rounds were the most important in determining the final score for the team, and the lowest score was discarded. The scores on ROI can be seen as a percentage score (similar to other simulations, see e.g., Mathieu and Rapp, 2009), and varied from −0.46 to 0.17, *M* = 0.03, *SD* = 0.11. In addition to the team score, each individual role within the team received an individual score. These individual scores did not count toward the team score, but did allow participants to compare their performance to their peers in other (competing) teams.

#### Control Variables

Control variables were age, gender, supply chain management knowledge (“How much knowledge do you have about supply chain management”; 1 = *very little*, 5 = *a lot*), prior experience with management simulations (“How experienced are you in playing management games”; 1 = *not at all*, 5 = *very experienced*), and number of hours per week spent on the game.

## Results

### Data Aggregation

Our theory and measurement were aimed at the team level of analysis, with the dependent variable of interest being a team-level variable, namely team performance expressed as ROI. Although in the current study individuals were nested within groups, multilevel techniques were not applied, as for these types of analyses the dependent variable needs to be at the lowest level of analysis (in this case the individual level; Bryk and Raudenbush, 1992). Although individual level scores were provided in the game, these scores did not determine the final group-level outcomes, as cross-functional integration and a clear strategy were key for performance in the game. Because the present study focused on a group-level dependent variable (i.e., team performance), aggregation to the group level is the most appropriate strategy to analyze the data (Kashy and Kenny, 2000). As presented above, the ICC(1) value and the rwg_(j)_ value were sufficient to justify aggregation (James et al., 1984, 1993; Bliese, 2000). Since the ICC(2) value also depends on team size, with higher values of ICC(2) as team size increases (Bliese, 2000), we chose to depend mainly on the outcomes of ICC(1) in deciding whether or not to aggregate the individual-level scores. We therefore used the mean (i.e., the average; see also Barrick et al., 1998) of the team members’ scores to represent shared task representations at the team level. This was not the case for majority decision-making, and team leadership ambiguity, as these had discrete answer categories, and, therefore, not a relative score.

### Descriptive Statistics

As can be seen in Table 1, age is positively related to experience (*r* = 0.20, *p* < 0.05), knowledge of supply chain management (SCM) (*r* = 0.27, *p* < 0.05), shared task representations (*r* = 0.31, *p* < 0.01), and team performance (*r* = 0.20, *p* < 0.05). Gender is negatively related to SCM knowledge (*r* = −0.31, *p* < 0.01). Also, the hours spent on playing the game are positively related to shared task representations (*r* = 0.18, *p* < 0.05), but not significantly positively related to team performance (*r* = 0.13, *ns*). Teams with a lot of SCM knowledge seemed to opt for majority decision-making slightly less (*r* = −0.21, *p* < 0.05), possibly because it was easier for them to reach a consensus decision. Finally, shared task representations are positively related to team performance (*r* = 0.23, *p* < 0.05), while the extent to which teams opt for majority decision-making is negatively related to team performance (*r* = −0.22, *p* < 0.05).

**TABLE 1:** Means, Standard Deviations, and Aggregate Level Intercorrelations.

*Variable*	*M*	*SD*	1	2	3	4	5	6	7	8	9
1. Age	33.61	8.59	–								
2. Gender	1.50	0.50	–0.10	–							
3. Hours spent	4.24	2.21	–0.01	0.12	–						
4. Management simulation experience	2.17	0.79	0.20*	–0.14	–0.15	–					
5. SCM knowledge	3.56	0.77	0.27*	−0.31**	–0.07	0.51***	–				
6. Shared task representations	3.84	0.41	0.31**	–0.00	0.18*	0.01	0.14	–			
7. Majority decision-making	0.10	0.20	–0.07	–0.03	0.04	–0.06	−0.21*	–0.12	–		
8. Leadership ambiguity	0.35	0.31	0.00	–0.00	–0.16	–0.03	0.03	0.03	0.06		
9. Team performance (ROI)	0.08	0.13	0.20*	0.06	0.13	0.09	–0.13	0.23*	−0.22*	–0.10	–

N = 81 teams; *p < 0.05, **p < 0.01, ***p < 0.001; two-tailed. SCM, Supply Chain Management; ROI, return on investment.

### Hypothesis Tests

**FIGURE 3:**
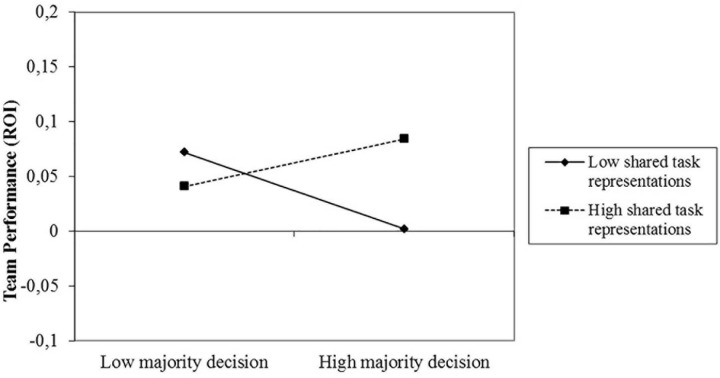
Team performance (ROI) as predicted by majority decision-making and shared task representations.

To test our hypotheses, we conducted hierarchical regression analysis with team performance as the dependent variable. Prior to the analyses, all continuous independent variables were mean-centered and the interaction terms as well as the main effects were based on the centered variables (Aiken and West, 1991). We also controlled for several variables that could potentially relate to team performance (i.e., age, gender, supply chain management knowledge, prior experience with management simulations, and number of hours per week spent on the game) and entered them into the equation at Step 1. At Step 2 we entered the three main effect terms (majority decision-making, shared task representations, and leadership ambiguity), at Step 3 the three two-way interactions, and at Step 4 the three-way interaction (see Table 2). Step 1 did not explain a significant proportion of variance in team performance and neither did Step 2. Thus, contrary to our expectations, we did not find support for hypothesis 1 stating that there should be a negative relationship between majority-decision making and team performance (β = −0.17*; ns).* However, Step 3 did explain a significant proportion of variance in team performance and revealed our predicted two-way interaction between majority decision-making and shared task representations (β = 0.25; *p* < 0.05; see Figure 3). To further analyze the interaction, we conducted simple slopes analyses (Aiken and West, 1991) and determined the simple slopes for teams with high and low shared task representations separately. As predicted, majority decision-making yielded a positive relationship to team performance for teams with higher levels of shared task representations (1 *SD* above the mean; *t* = 2.71, *p* < 001), and a negative relationship for teams with lower levels of shared task representations (1 *SD* below the mean; *t* = −5.01, *p* < 0.001).

**TABLE 2:** Hierarchical Regressions with Dependent Variable Team Performance (ROI).

	Step 1		Step 2		Step 3		Step 4	
Variable	β	SE	β	SE	β	SE	β	SE
***Control Variables***								
Age	0.16	0.00	0.12	0.00	0.03	0.00	0.05	0.00
Gender	0.12	0.03	0.10	0.03	0.01	0.03	–0.02	−0.01
Hours spent	0.13	0.01	0.10	0.01	0.07	0.01	0.12	0.01
Management simulation exp.	–0.01	0.02	0.01	0.02	0.11	0.02	0.16	0.02
SCM knowledge	0.19	0.02	0.13	0.02	–0.00	0.02	–0.03	0.00
***Main effects***								
Majority decision-making			–0.17	0.07	–0.12	0.06	–0.05	0.06
Shared task representations			0.13	0.04	0.23*	0.03	0.14	0.03
Leadership ambiguity			–0.07	0.05	0.04	0.04	0.07	0.04
***Interaction 2-way***								
MDMxSTR					0.36**	0.23	0.25*	0.23
MDMxLA					–0.14	0.22	–0.08	0.21
STRxLA					0.15	0.11	0.16	0.11
***Interaction 3-way***								
MDMxSTRxLA							0.32**	0.54
*R*^2^	0.09		0.14		0.36		0.42	
Δ*R*^2^	0.09		0.05		0.21		0.06	
Δ*F*	1.47		1.49		7.70***		7.15*	
*dfs*	(5, 75)		(3, 72)		(3, 69)		(1, 68)	

N = 81 teams; *p < 0.05; **p < 0.01; ***p < 0.001; two-tailed; Total *R* = 0.65 for step 4; SCM, Supply Chain Management; MDM, Majority decision-making; STR, Shared task representations; LA, Leadership ambiguity; ROI, return on investment.

**FIGURE 4:**
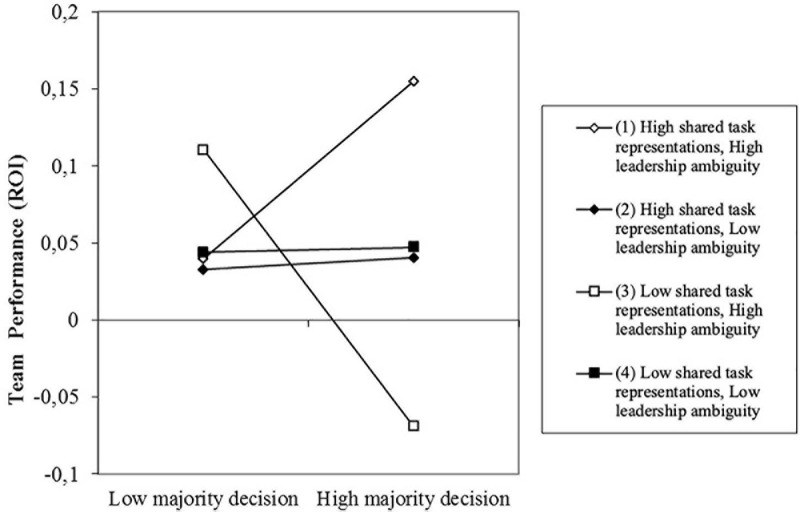
Team performance (ROI) as predicted by majority decision-making, shared task representations and leadership ambiguity.

Moreover, Step 4 did explain an additional significant proportion of variance in team performance and revealed our predicted three-way interaction between majority decision-making, shared task representations and leadership ambiguity (β = 0.32, *p* < 0.01; see Table 2, and Figure 4). Visual inspection of the figure indicates that, as expected, team performance is highest for teams using a majority rule, who also have high levels of shared task representations and high leadership ambiguity, whereas it is lowest for teams using a majority rule, who have low levels of shared task representations and high leadership ambiguity. To further analyze the interaction, we conducted simple slopes analyses (Aiken and West, 1991). As predicted, majority decision-making yielded a marginally significant positive relationship to team performance for teams with high task representations and high leadership ambiguity (*t* = 1.85, *p* = 0.07; see slope 1 in Figure 4) and a significant negative relationship to team performance for teams with low shared task representations and high leadership ambiguity (*t* = −4.56, *p* < 001; see slope 3 in Figure 4). As expected, the slopes for high task representations/low leadership ambiguity (*t* = 0.13, *ns*; see slope 2 in Figure 4) and for low task representations/low leadership ambiguity (*t* = 0.04, *ns;* see slope 4 in Figure 4) were not significant. In addition, we calculated slope difference tests for all six pairs of slopes (Dawson and Richter, 2006). These allow for comparative tests between sets of slopes, as opposed to the absolute tests of single slopes calculated by the simple slope analyses presented above (Dawson, 2014). These tests indicated that that there are significant differences for three pairs of slopes. The difference between slope 1 (high shared task representation/high leadership ambiguity) and slope 3 (low shared task representation/high leadership ambiguity) was significant (*t* = 3.88, *p* < 0.001), which is in line with our expectation that high leadership ambiguity should strengthen the positive (vs.) negative effects of shared task representations on team performance, under conditions of high majority decision-making. The difference between slope 2 (high shared task representation/low leadership ambiguity) and slope 3 (low shared task representation/high leadership ambiguity) was also significant (*t* = 2.35; *p* < 0.05), and finally the difference between slope 3 (low shared task representation/high leadership ambiguity) and 4 (low shared task representation/low leadership ambiguity) was also significant (*t* = −2.73; *p* < 0.01). Overall, it seems that the combination of low shared task representation with high leadership ambiguity differed significantly from all other slopes.

## Discussion

Decision-making groups working on a complex interdependent task with uniquely distributed information often do not make optimal use of their informational resources (Stasser and Birchmeier, 2003) and the decision rules that teams use may affect the way this information is processed. Since majority decision-making is the most prevalent decision rule used in intact groups and its effects on team performance in complex interdependent tasks have been shown to be inconsistent (cf. Beersma and De Dreu, 2002; Ten Velden et al., 2007), it is important to identify under what conditions it may lead to better quality decisions and performance. First, we argued that, in complex tasks with distributed information, the majority decision-making rule should be negatively related to team performance, however, we did not find support for this prediction. Second, we argued that, whereas the decision rule used by the team may be of utmost importance in predicting performance, it cannot be seen in isolation from other aspects of group processes, such as the development of shared task representations and the extent to which there is clarity about who is responsible for leadership in the team. To this end, we argued that the effects of majority decision-making on team performance may be contingent on the extent to which teams have developed a shared understanding that success is predicated on the elaboration and integration of information distributed among team members (i.e., shared task representations). Indeed, as predicted, we found that majority decision-making was positively related to team performance for teams with high shared task representations and negatively related to team performance for teams with low shared task representations. Finally, we argued and found that majority decision-making, shared task representations and leadership ambiguity interact in predicting team performance. Specifically, we expected that leadership ambiguity would strengthen the positive (vs. negative) effects of shared task representations on team performance, for those teams favoring a majority decision-rule. As predicted, we found that a majority decision-making rule was positively related to performance for those teams who had high task representations and high leadership ambiguity, whereas it was negatively related to performance for those teams who had low task representations and high leadership ambiguity. In addition, the significant difference between the high shared task representations/high leadership ambiguity slope and the low shared task representations/high leadership ambiguity slope, suggests that leadership ambiguity does indeed strengthen the positive (vs.) negative effects of shared task representations on team performance, under conditions of high majority decision-making. Interestingly, the slope for low shared task representations/high leadership ambiguity differed significantly from all other slopes, suggesting that it was the particular combination of low shared task representations, high leadership ambiguity and a majority decision-making rule that harmed team performance. Finally, as expected, we also found that under conditions of low leadership ambiguity, the effects of shared task representations on team performance were less pronounced for teams using a majority rule. In conclusion, it appears that, for teams favoring a majority decision-making rule, the combination of high shared task representations and high leadership ambiguity is particularly beneficial for team performance, whereas the combination of low shared task representation and high leadership ambiguity is particularly harmful for performance.

The substantive contributions of the current study are twofold. First, we extend existing theory on decision rules by showing that a majority rule is more effective in combination with shared task representations. Second, we build on the emerging literature on emerging and shared leadership by showing that, under some circumstances, leadership ambiguity can be beneficial for team performance. While it has previously been reasoned that a clear leader is imperative in providing a compelling direction and in ensuring clarity of and commitment to team objectives (West et al., 2003), the current study shows that when teams have a compelling sense of direction in terms of shared task representations, leadership clarity can actually be detrimental for team performance when majority decision-making is high.

### Theoretical and Practical Implications

Prior research has found that clarity of leadership was especially important for larger teams in terms of innovation, probably because, in such teams, having a clear team leader prevented loss of coordination (West et al., 2003). Although a transformational team leader can indeed play a role in developing a shared vision and, in turn, promote team reflexivity (Schippers et al., 2008), the current study shows that under conditions of high majority decision-making, leadership ambiguity can be beneficial when shared task representations are also high. In other words, leadership ambiguity can be beneficial if teams have developed a shared understanding of what it takes to be successful and have opted for an equality-based majority decision rule. Managers should therefore consider under which circumstances the “leader decides” rule should apply, and under what conditions the majority rule might be more beneficial (cf. Hastie and Kameda, 2005). For instance, if teams decide to use a majority rule and they have a shared understanding of what the task entails, they would benefit from having a manager or leader that is less prominent or even absent.

Theoretically, it should be noted that authority differentiation, or the extent to which all team members are involved in team decision-making processes (Hollenbeck et al., 2012), has some similarities to majority decision-making. However, in the context of the current paper, we were especially interested in the rules that teams use to make decisions. Thus, while authority differentiation can be related to the process of decision-making, and the extent to which team members are involved in the process, teams can still choose a specific decision rule to make the actual decision. Future research could, therefore, focus on the role of authority differentiation that precedes decision-making.

### Limitations and Future Directions

Whereas an obvious strength of the current study is that we tested our hypotheses with a large number of teams, comprising mainly of professionals in a realistic setting, we should recognize that only experimental studies can speak to the causality implied in the research model. A clear direction for future research would thus be to replicate these findings by using experimental designs and manipulating decision rules, shared task representations and leadership ambiguity.

A limitation of sorts is that while we do indeed have evidence of the core team processes and decision rules involved – majority decision-making, shared task representations, and leadership ambiguity – it is not completely clear how these played out in practice. That is, we do not know exactly what happened in teams with leadership ambiguity, and whether in teams with leadership ambiguity there was indeed more room for elaboration of task-relevant information. Furthermore, elaboration of information might also have taken place more implicitly, as team members could also elaborate information as a habitual practice without conscious, or explicit awareness. Another question is whether teams performing well in the game, also perform well in the real world. For instance, we controlled for levels of experience in the field of supply change management to address the fact that some of our teams had little experience. While evidence in this respect is not required for the test of our hypotheses – nor is any specific content suggested by our analysis – such information could be extremely helpful in further developing our analysis, as it may provide key pointers as to as to what factors influence the effectiveness of majority decision-making. Future research to address this issue would therefore be very valuable.

Also, it should be noted that none of the teams reported conflict over leadership. While an earlier study found leadership ambiguity to be a combination of “there is no clear leader/coordinator” and “there is conflict over who leads/coordinates the team” (West et al., 2003), in the current study, this variable denoted solely the absence of a clear leader/coordinator, since none of the team members indicated conflict over leadership. Hence, this might explain the differences in results between our study and the one by West et al. (2003). Whereas they found that leadership ambiguity was negatively related to team processes and team innovation, we did not find leadership ambiguity to be directly related to performance. The absence of conflict over leadership in our teams may explain this difference. In addition, the dependent variable in the West et al. (2003) paper was team innovation, which is conceptually different from team performance. Future research could investigate in how far the effects of leadership ambiguity may differ for team innovation and team performance.

Another limitation has to do with the reporting of moderated multiple regression (MMR). Recent theorizing suggests that these analyses often report small effect sizes and are often underpowered (Murphy and Russell, 2017). A 20-year review noted that outcome reporting bias may play a role, especially if sample sizes are small, and/or the p value is just below the.05 threshold (O’Boyle et al., 2019). In the current paper, neither of these were the case, therefore increasing our confidence in our results. Nevertheless, we cannot be certain that this is not a type II error. Furthermore, although we did hypothesize the relationships with respect to the two- and three ways interactions before-hand, we also used a combination of *a priori* reasoning and abduction (“a form of reasoning that moves from observations in a specific situation, information source, or data set to an explanation that accounts for those particular observations”; Behfar and Okhuysen, 2018, p. 325). Therefore, it is important for future research to replicate our findings. Also, we need to acknowledge some limitations with respect to common method bias, since all independent variables were self-reported and assessed at the same time (cf. Podsakoff et al., 2003), which could lead to an overestimation of the main effects. However, it is also important to note that common source or method bias cannot account for statistical interactions. Because it may inflate the main effects it may lead to an underestimation of effect sizes for interactions (Evans, 1985; McClelland and Judd, 1993). It should also be noted that we assessed the outcome measure at a later point in time. Another potential limitation we need to acknowledge is that our ICC(2) value for shared task representations was not as high as the usually recommended cut-off value of.80 (Van Mierlo et al., 2009), which may raise some questions regarding the extent to which shared task representations do indeed represent a shared construct (LeBreton and Senter, 2008). However, it is also worth noting that the ICC(2) value depends on team size, with higher values of ICC(2) as team size increases (Bliese, 2000), and that the ICC(1) and the rwg_(j)_ value were deemed sufficient to justify aggregation (James et al., 1984, 1993; Bliese, 2000). Nevertheless, this is something to be further investigated in future research.

Finally, we did not formally model any time-sensitive mediating or moderating models that might have accounted for the observed relationships (cf. Mathieu and Rapp, 2009). Future research could benefit from measuring the core process variables (majority decision-making, task representations and leadership ambiguity) on a weekly basis and use growth modeling to see whether the model holds up over time, and to identify the dynamics over time (e.g., Bliese et al., 2007; Ployhart and Vandenberg, 2010).

### Conclusion

The current study integrates and extends theorizing on the relationship between decision rules and team processes. Since the use of decision rules can greatly influence team processes and outcomes (e.g., Hastie and Kameda, 2005), it is imperative to understand the contingencies influencing the relationship between decision rules and team performance. Our analysis has shown that the relationship between majority decision-making and performance is not a simple one. The effectiveness of the majority decision-making rule is contingent on both shared task representations and leadership ambiguity. Thus, to make optimal use of the majority decision rule in complex tasks, teams would benefit from developing shared task representations, emphasizing information elaboration, and from operating under conditions of high leadership ambiguity.

## Data Availability Statement

The datasets generated for this study are available on request to the corresponding author.

## Ethics Statement

Ethical review and approval was not required for the study on human participants in accordance with the local legislation and institutional requirements. The patients/participants provided their written informed consent to participate in this study.

## Author Contributions

Both authors provided substantial contributions to the conception or design of the work, were responsible for drafting the work or revising it critically for important intellectual content, approved the final version of this manuscript, and agreed to be accountable for all aspects of the work.

## Conflict of Interest

The authors declare that the research was conducted in the absence of any commercial or financial relationships that could be construed as a potential conflict of interest.

## Footnotes

1 https://www.thefreshconnection.biz/

2 Two other options, added by the game provider, were “We argued a lot about the decisions we had to make” and “We often agreed quickly.” Hardly any teams opted for these, and adding these options as control variables did not change our pattern of results. In addition, we checked for the correlations between the answer options. Not surprisingly, there was a relatively high correlation between the unanimity rule and the majority rule (*r* = −0.513 *p* < 0.001). The relationship between majority decision-making and one dominant team member making all decisions was rather low, and not significant (*r* = −0.124; ns). However, controlling for these other two options in the regression analysis (i.e., the unanimity rule and a dominant group member making decisions; see Appendix for the items) did not change the pattern or significance of our results.

3 Note that in the West et al. (2003) paper, leadership clarity was calculated by the proportion of respondents who either said: “There is no clear leader/coordinator” or “There is conflict over who leads/coordinates the team.” Since none of the team members in our dataset indicated that there was conflict over who was leading the team, leadership ambiguity was calculated to represent the proportion of team members indicating that there was no clear leader/coordinator. In addition, we checked for the correlations between the answer options. As expected, the extent to which teams indicated that “There is a very clear leader” was negatively related to leadership ambiguity (*r* = −0.616, *p* < 0.001). However, controlling for the other answer options in the regression analysis (i.e., there is a clear leader, a number of people are leading, we all had coordinating roles; see Appendix for the items), did not change the pattern or significance of our results.

## Appendix: Measures used

**Shared task representations**

1. For high quality performance it was important to base the decision on as much information as possible.
2. Strategy discussions among team members were crucial for high performance.
3. Discussing all members’ information was of crucial importance for attaining high decision quality on this task.
4. I had the impression the other team members would appreciate discussion.
5. I expected my team members to be open for critics and allow for critical discussions to take place.

**Decision-making rules/process**

*How were decisions made in your team?*

◻ One dominant team member made most of the decisions
◻ All decisions were made as a team
◻ We had a majority rule

**Leadership ambiguity**

*Was there a clear overall leader in your team?*

◻ There was a single very clear leader/coordinator
◻ A number of people lead/coordinated the team
◻ There was no clear leader/coordinator
◻ There was conflict over who leads/coordinates the team
◻ We all had leadership/coordinator roles

**Control variables items**

– How much knowledge do you have about supply chain management?
– How experienced are you in playing management games?
– How much time did you spend playing the game? ………. Hours per week.
